# Functional characterization and evolution of the isotuberculosinol operon in *Mycobacterium tuberculosis* and related Mycobacteria

**DOI:** 10.3389/fmicb.2012.00368

**Published:** 2012-10-12

**Authors:** Francis M. Mann, Meimei Xu, Emily K. Davenport, Reuben J. Peters

**Affiliations:** Department of Biochemistry, Biophysics, and Molecular Biology, Iowa State UniversityAmes, IA, USA

**Keywords:** isoprenoid biosynthesis, molecular evolution, virulence, terpenoids

## Abstract

Terpenoid metabolites are important to the cellular function, structural integrity, and pathogenesis of the human-specific pathogen *Mycobacterium tuberculosis* (*Mtb*). Genetic and biochemical investigations have indicated a role for the diterpenoid isotuberculosinol (isoTb) early in the infection process. There are only two genes (Rv3377c and Rv3378c) required for production of isoTb, yet these are found in what appears to be a five-gene terpenoid/isoprenoid biosynthetic operon. Of the three remaining genes (Rv3379c, Rv3382c, and Rv3383c), previous work has indicated that Rv3379c is an inactive pseudo-gene. Here we demonstrate that Rv3382c and Rv3383c encode biochemically redundant machinery for isoprenoid metabolism, encoding a functional 4-hydroxy-3-methylbut-2-enyl diphosphate reductase (LytB) for isoprenoid precursor production and a geranylgeranyl diphosphate (GGPP) synthase, respectively, for which the *Mtb* genome contains other functional isozymes (Rv1110 and Rv0562, respectively). These results complete the characterization of the isoTb biosynthetic operon, as well as further elucidating isoprenoid metabolism in *Mtb*. In addition, we have investigated the evolutionary origin of this operon, revealing *Mtb*-specific conservation of the diterpene synthase genes responsible for isoTb biosynthesis, which supports our previously advanced hypothesis that isoTb acts as a human-specific pathogenic metabolite and is consistent with the human host specificity of *Mtb*. Intriguingly, our results revealed that many mycobacteria contain orthologs for both Rv3383c and Rv0562, suggesting a potentially important role for these functionally redundant GGPP synthases in the evolution of terpenoid/isoprenoid metabolism in the mycobacteria.

## INTRODUCTION

Each year, *Mycobacterium tuberculosis* (*Mtb*) accounts for ~1.7 million deaths as the main causative agent of the human disease Tuberculosis (Rohde et al., 2007), and the ability of this deadly pathogen to infiltrate macrophage cells from the mammalian immune system remains an active area of investigation (Russell, 2007). While the complete pathogenic mechanism remains unknown, the bacteria appear to launch a multi-factorial attack (Russell et al., 2010), with many bacterial genes involved in pathogenesis (Joshi et al., 2006). Notably, a mutational screen for genes involved in the early infection process highlighted a role for Rv3377c and Rv3378c, loss of which reduced the ability of *Mtb* to block acidification of the engulfing phagosome compartment, leading to suppressed bacterial proliferation in macrophage cell cultures (Pethe et al., 2004). These genes have since been characterized as encoding a pair of sequentially acting diterpene synthases responsible for the production of isotuberculosinol (isoTb; Nakano et al., 2005a,b; Mann et al., 2009b; Maugel et al., 2010; Prach et al., 2010; Spangler et al., 2010; Hoshino et al., 2011; Mann et al., 2011b; Nakano et al., 2011), a diterpenoid capable of arresting phagosomal maturation *in vitro* (Mann et al., 2009b). Thus, isoTb appears to play an immunomodulatory role in the *Mtb *infection process (Mann and Peters, 2012).

**FIGURE 1:**
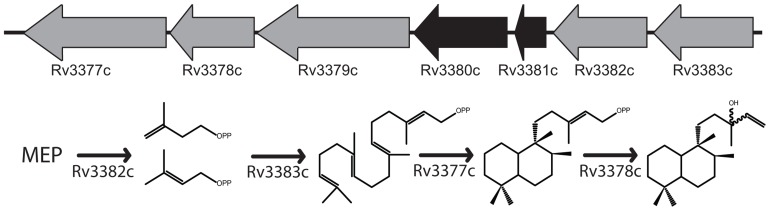
**Proposed isoTb biosynthetic operon**. The five-gene operon including the two previously characterized diterpene synthases required for isoTb biosynthesis and the proposed roles of the remaining uncharacterized genes in the biosynthetic pathway. Genes depicted in the order suggested by their numbering rather than orientation of open reading frame.

Rv3377c and Rv3378c fall within a larger operon that appears to have been acquired via horizontal gene transfer before the divergence of *Mtb* and *Mycobacterium bovis* (Becq et al., 2007). The operon includes Rv3377c–Rv3383c, with the intervening Rv3380c and Rv3381c representing apparent transposable elements inserted into a preexisting operon (Camus et al., 2002), such that only five genes seem to be relevant. By homology, these all appear to encode enzymes that could be involved in various stages of isoTb production (**Figure 1**). However, only Rv3377c and Rv3378c were isolated in the genetic screen targeting the early infection process, despite the saturating nature of that study (Pethe et al., 2004). The remaining genes do not appear to be required for any essential cellular function, as indicated by previously reported genetic screens (Sassetti et al., 2001, 2003; Sassetti and Rubin, 2003; Joshi et al., 2006), leaving uncertain their function, if any, in mycobacterial terpenoid biosynthesis. It has already been reported that Rv3379c is an inactive pseudo-gene (Bailey et al., 2002), leaving the function of the remaining genes in question.

*Mycobacterium tuberculosis* uses the methyl-erythritol-5-phosphate (MEP) pathway for isoprenoid precursor biosynthesis (Rohmer et al., 1993). The resulting 5-carbon isoprenyl isomers, isopentenyl diphosphate (IPP) and dimethylallyl diphosphate (DMAPP), are then coupled together to form longer acyclic isoprenyl diphosphates such as the 10-carbon geranyl diphosphate (GPP), 15-carbon farnesyl diphosphate (FPP), and 20-carbon diterpenoid precursor geranylgeranyl diphosphate (GGPP). These are then used in cell wall assembly (e.g., the further elongated decaprenyl diphosphate), synthesis of other essential biomolecules (e.g., menaquinone), as well as isoTb biosynthesis. In the isoTb operon, Rv3379c is homologous to the deoxy-xylulose-5-phosphate synthases that initiate the MEP pathway, and is annotated as *dxs2*, although it has been reported to be an inactive pseudo-gene (Bailey et al., 2002). Rv3382c encodes a putative 4-hydroxy-3-methylbut-2-enyl diphosphate reductase, the LytB that catalyzes the final step of the MEP pathway, producing IPP and DMAPP (Rohdich et al., 2002), but this has not previously been investigated. Rv3383c encodes a member of the isoprenyl diphosphate synthase (IDS) family, which generally catalyze isoprenyl diphosphate chain elongation. Accordingly, the IDS encoded by Rv3383c is likely to produce the GGPP required for isoTb biosynthesis, but this also has not yet been investigated. Here, we not only verify the biochemical function of both Rv3382c and Rv3383c, but also the presence of robust isozymes catalyzing the same reactions encoded elsewhere in the *Mtb* genome, suggesting functional redundancy consistent with the reported genetic screens. In addition, bioinformatics analysis of the available mycobacterial genome sequences indicates asymmetric conservation of isoTb biosynthetic machinery in *Mtb* relative to the greater *Mycobacterium tuberculosis* complex (MTC), consistent with a previously hypothesized role for isoTb in the highly infectious nature of *Mtb* in humans, along with the surprising finding of wider conservation of dual GGPP synthases in a sub-group of mycobacteria.

## MATERIALS AND METHODS

### GENERAL

All *trans* (*E*) isomers of GPP, FPP, and GGPP were purchased from Isoprenoids, LC (Tampa, FL, USA). Unless otherwise noted, all other chemicals were purchased from Fisher Scientific (St. Louis, MO, USA) and all molecular biology reagents were purchased from Invitrogen (Carlsbad, CA, USA). Gas chromatography-flame ionization (GC-FID) detection was carried out using an Agilent 6890 GC-FID (Agilent Technologies, Santa Clara, CA, USA) using a previously described method (Schmidt and Gershenzon, 2008).

### BIOINFORMATICS

Genes of interest were identified via basic local sequence alignment (BLAST) against the *M. tuberculosis* H37Rv genome (Cole et al., 1998). Phylogenetic analysis of GGPP synthases (GGPPS) was accomplished via UPGMA modeling of amino acid sequences (Sneath and Sokal, 1973), using CLC Sequence Viewer 6.0 (CLC Bio, Cambridge, MA, USA). Complete operon analysis was performed against publically available sequences of *Mtb* diversity strains (Hershberg et al., 2008), *M. canettii* (Hershberg et al., 2008), *M. marinum* (Stinear et al., 2008), and *M. ulcerans* (Stinear et al., 2007), as well as previously published sequences of *M. bovis* (Garnier et al., 2003), and *M. bovis* BCG (Brosch et al., 2007), and the pre-publication reads of *M. africanum* (available via the Sanger Institute). Genomic regions were aligned using BLAST analysis and synteny mapping via the Tuberculosis Database (Reddy et al., 2009).

### CLONING

Genes of interest were cloned by PCR amplification from *Mtb* CDC1551 genomic DNA and confirmed via complete sequencing. These were transferred into pENTR/SD/D-TOPO (Gateway, Invitrogen) by directional recombination. Rv2173 and Rv0562 were modified to change the first codon to ATG to enable expression in *Escherichia coli*, for which purpose all genes were recombined into pDEST17 expression vectors for production with an N-terminal 6xHis tag.

### LytB COMPLEMENTATION

LytB complementation was initially assessed by colony counting assays, carried out in three individual experiments, each in duplicate. 50 ng of plasmid (pDEST14) containing Rv3382c or Rv1110 were each electroporated into 100 μL electro-competent *E. coli* cells containing LytB under arabinose promoter control (McAteer et al., 2001) and were plated on NZY medium in the absence of arabinose, which allows only for growth of LytB complemented cells. Electroporated *E. coli* cells plated in the presence and absence of 0.2% arabinose were used as controls. More quantitative analysis of LytB activity was accomplished by comparison of relative growth rates, carried out in triplicate. Colonies from Plasmid (+) plates were grown to saturation in LB medium overnight. Cultures were diluted 1/100 into fresh LB medium containing 0.5 mM isopropylthiogalactoside (IPTG) and grown for 1 h, then diluted 1/50 into media containing 50 μg/mL carbenicillin and 0.2% (v/v) glucose to inhibit the arabinose promoter and thus completely inhibit any expression of *E. coli* LytB. After 220 min, cultures were diluted 1/50 into medium containing 0.2% (v/v) arabinose, thus activating the chromosomal LytB and allowing for comparative analysis of the relative health of both complemented strains (i.e., to demonstrate that expression of Rv3382c did not exert any toxicity relative to expression of Rv1110 instead). Culture growth was monitored every 20 min by measuring OD_600_.

### PUTATIVE GGPP SYNTHASE EXPRESSION

Putative GGPP synthases in pDEST17 were transformed into the C41 strain of *E. coli* (Lucigen, Middleton, WI, USA), and plated on NZY agar with carbenicillin (50 μg/mL) selection. Colonies were inoculated in 50 mL of NZY medium and grown to an optical density at 600 nm (OD_600_) of 0.6 at 37^°^C before being transferred to 16^°^C for 1 h prior to induction with 0.5 mM IPTG, with subsequent 14–16 h incubation. Cells were harvested via centrifugation and lysed in 1 mL lysis buffer (10 mM Tris-Cl, pH 6.8, 10% glycerol, 1 mM dithiothreitol) via sonication. These lysates were clarified via centrifugation (10 min × 10,000*g*) and the resulting cell free extracts assayed for GGPP synthase activity. As *E. coli *does not harbor contaminating GGPP synthases (Burke and Croteau, 2002), any resulting production of GGPP was attributed to expression of the recombinant gene.

### GGPP SYNTHASE ASSAY

Assay buffer (50 mM sodium phosphate (pH 7.0), 10% glycerol, 5 mM MgCl_2_, 1 mM dithiothreitol) was mixed with 20 μM IPP and the equivalent concentration of indicated allylic isoprenyl diphosphate. To each assay, 100 μL of purified protein (~1 mg/mL) expressed as a 6×His fusion in pDEST17 was added, totaling 1 mL total volume. Assays were conducted at 37^°^C for indicated time and halted via incubation at 100^°^C for 5 min. After cooling, 1.5 U calf intestinal alkaline phosphatase (New England Biolabs, Ipswich, MA, USA) was added according to manufacturer protocol. The phosphatase reaction was incubated overnight at 37^°^C and extracted thrice with 1 mL hexanes. Samples were concentrated and analyzed via GC-FID and compared to similarly, dephosphorylated authentic GGPP.

### KINETIC ANALYSIS OF Rv3383c AND Rv0562

The 6×His tagged versions of these enzymes were purified via Ni-NTA Superflow resin (Novagen, Merck, Germany) following the manufacturer directions. Protein was analyzed via denaturing gel electrophoresis (SDS-PAGE), demonstrating >90% purity. Enzymes were assayed as described against a substrate gradient (0–40 μM) with IPP and DMAPP/FPP for 5 min with 100 nM enzyme. Post-extraction, cembrene was added to a final concentration of 5 μM as an internal standard for quantification. Product concentration was calculated by integration of the product peak area and comparison to internal standard peak area. Michaelis–Menten curves were fit to the observed data (KaleidaGraph 4.0, Synergy Software, Reading, PA, USA), resulting in *R*^2^ values ≥0.95.

## RESULTS

### POTENTIAL ISOZYMES FOR BOTH Rv3382c AND Rv3383c

To begin investigating the hypothesized biochemical redundancy, the uncharacterized Rv3382c and Rv3383c were analyzed and compared to the rest of the *Mtb* genome. Rv3382c is 990 bp long and encodes a protein of 329 amino acids (AA) that has been annotated as *lytB1*, while Rv3383c is 1053 bp long and encodes a 350 AA protein annotated as *idsB* (Camus et al., 2002). Both genes are non-essential (Sassetti et al., 2003), and neither is associated with pathogenicity in published genetic screens (Sassetti and Rubin, 2003; Pethe et al., 2004; Fortune et al., 2005; Joshi et al., 2006). Protein–protein BLAST analysis of Rv3382c and Rv3383c against the H37Rv genome revealed putative paralogs for both. Specifically, Rv3382c shares 50% AA identity with Rv1110, which is similarly annotated as *lytB2* (Camus et al., 2002). There are four IDS paralogs to Rv3383c in the *Mtb* genome: Rv3398c, a previously identified FPP synthase annotated as *idsA1* (Dhiman et al., 2004), which shares 35% AA identity with Rv3383c; Rv2173, annotated as *idsA2*, which shares 29% AA identity; Rv0562, annotated as*grcC1*, which shares 30% AA identity; and Rv0989c, that we have recently demonstrated encodes a functional GPP synthase likely involved in decaprenyl diphosphate biosynthesis (Mann et al., 2011a), annotated as *grcC2*, which shares 28% AA identity.

### FUNCTIONAL ANALYSIS OF Rv3382c AND Rv1110

**FIGURE 2:**
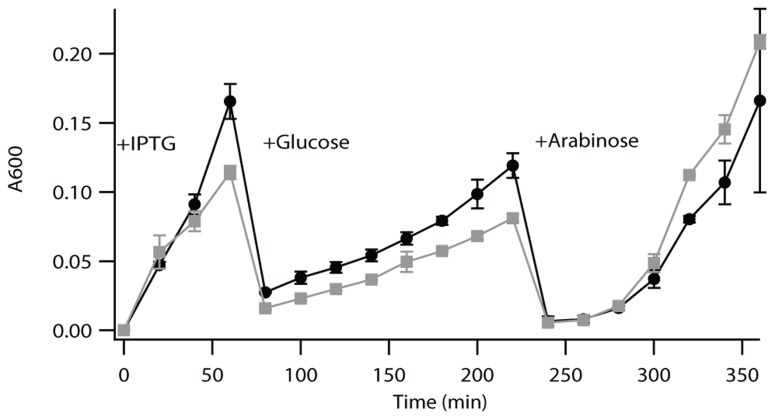
**Complementation assay for LytB activity**. Growth curve of lytB^-^ *E. coli* complemented with Rv1110 (circles) or Rv3382c (squares). These bacteria were grown to saturation, then diluted 1/100 into fresh media containing 0.5 mM IPTG. After 60 min, cultures were diluted 1/50 in media containing selective antibiotic and 0.02% glucose (to suppress endogenous LytB expression). After 220 min, cultures were diluted 1/100 in media containing selective antibiotic, but 0.2% arabinose instead of glucose, which activated expression of the endogenous LytB, to demonstrate that the Rv3382c gene itself did not have any deleterious effects (i.e., as both recombinant strains then exhibited equivalent growth).

LytB is a [4Fe–4S] protein that has been shown to be difficult to work with *in vitro*, due to complications from both oxygen corruption and substrate availability (Adam et al., 2002; Wolff et al., 2003). Accordingly, such enzymatic activity is most often demonstrated by a complementation approach (McAteer et al., 2001; Rohdich et al., 2002; Hsieh and Goodman, 2005). Briefly, this utilizes a previously developed strain of *E. coli* in which the endogenous LytB gene (EG11081) has been put under control of an arabinose promoter, such that bacterial growth requires either supplementation with arabinose or complementation with a functional LytB (McAteer et al., 2001). Both Rv3382c and Rv1110 were able to complement the growth of this strain of *E. coli* in the absence of arabinose, demonstrating functional LytB activity. Relative growth rate analysis indicates that complementation with Rv3382c results in somewhat less vigorous growth than does complementation with Rv1110, which does not appear to be due to a toxicity effect (**Figure 2**). This result then suggests that Rv3382c exhibits somewhat less robust LytB activity than Rv1110, which might be due to Rv3382c being 8 AA shorter at the N-terminus. However, this region does not contain any residues known to be involved in enzymatic activity, and the exact mechanism underlying reduced function of the LytB encoded by Rv3382c is unclear.

### ANALYSIS OF PUTATIVE GGPP SYNTHASES

**FIGURE 3:**
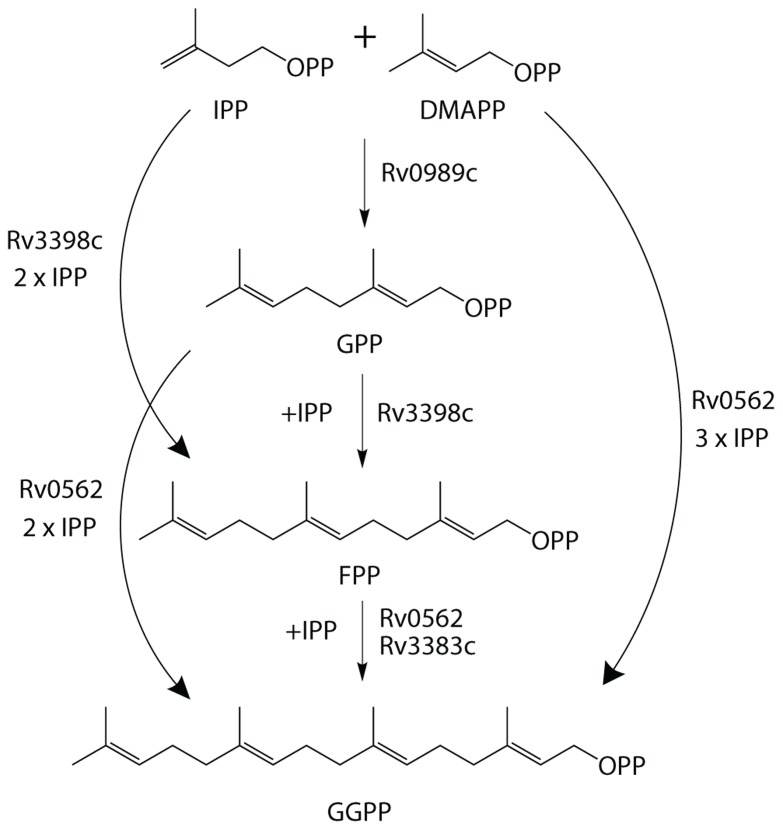
**Substrate plasticity of GGPPS**. GGPPS enzymes are sometimes able to utilize a variety of substrates to synthesize the 20-carbon product, GGPP. Outlined are the various biosynthetic pathways for GGPP production in *Mtb*.

**FIGURE 4:**
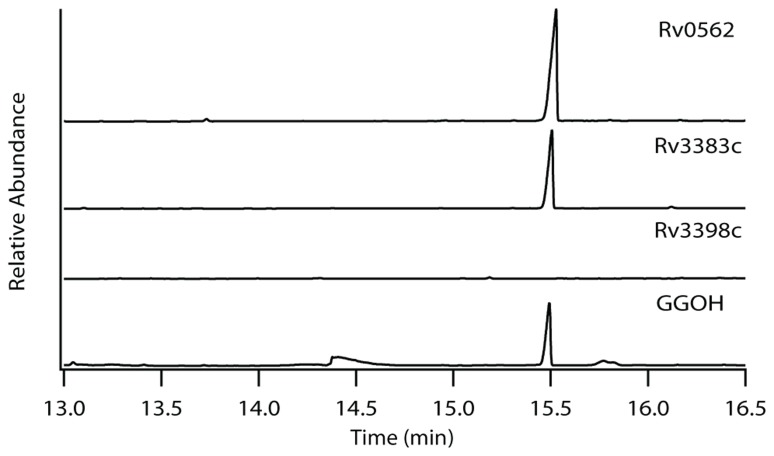
**Production of GGPP by Rv3383c and Rv0562**. GC-FID chromatogram of GGPP-derived GGOH production in Rv0562 and Rv3383c, but not the previously confirmed (*E,E*)-FPP synthase Rv3398c, as compared to dephosphorylated authentic standard.

The production of GGPP is catalyzed by the condensation of IPP with allylic isoprenyl diphosphate acceptors, such as DMAPP (which then requires the addition of three molecules of IPP), GPP (e.g., that produced by Rv0989c, which then requires the addition of two molecules of IPP), or FPP (e.g., that produced by Rv3398c, which then only requires the addition of a single molecule of IPP; **Figure 3**). Thus, the putative GGPP synthases identified above were screened for such activity by assaying purified recombinant proteins expressed in *E. coli*, in the presence of IPP plus DMAPP, GPP, or FPP (**Table 1**). Both Rv0562 and Rv3383c were able to add IPP to FPP, producing GGPP (**Figure 4**). In addition, Rv0562 is able to produce GGPP using either DMAPP, GPP, or FPP as the allylic precursor, while Rv3383c can synthesize GGPP only when fed FPP (**Table 1**). By contrast, Rv2173 only exhibited intermittent GGPP synthase activity in these assays, indicating that its primary product may not be GGPP. Further studies are ongoing with this enzyme to clarify its function.

**Table 1:** Kinetic analysis of confirmed GGPP synthases.

Enzyme	IPP	DMAPP		GPP		FPP	
	*K_M_* (μM)	*kcat* (min^-1^)	*KM* (μM)	*kcat* (min^-1^)	*KM* (μM)	*kcat* (min^-1^)	*KM* (μM)
Rv0562	1.7 ± 0.7	0.17 ± 0.02	5 ± 2	0.3 ± 0.2	2.3 ± 0.8	0.7 ± 0.1	11 ± 3
Rv3383c	11 ± 2	NR	NR	NR	NR	1.0 ± 0.5	19 ± 5

*NR, no reaction.*

### EVOLUTION OF THE isoTb OPERON WITHIN THE MYCOBACTERIA

**FIGURE 5:**
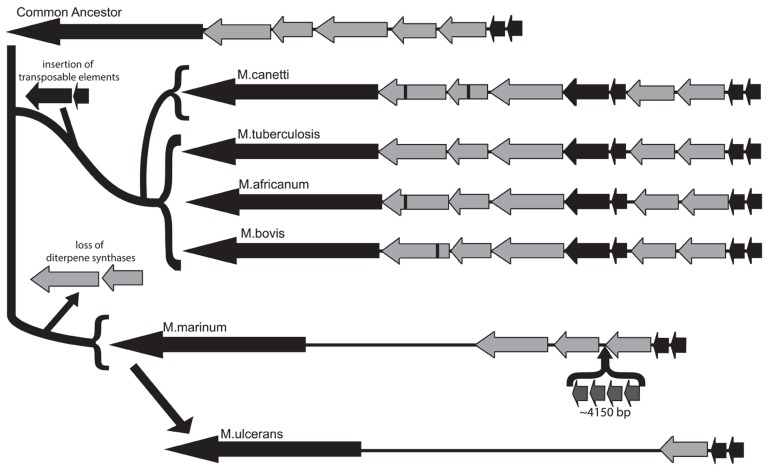
**Conservation of the isoTb operon in the MTC and closely related mycobacteria**. Analysis of the isoTb operon in the context of wider mycobacterial evolution indicates the operon was likely acquired in the most recent common ancestor to the MTC and *M. marinum*. The functional genes in the operon (light gray arrows) were retained during the speciation events leading to *M. canetti*, *Mtb*, *M. africanum*, and* M. bovis*, following which the operon independently evolved in each species, as evidenced by unique insertion–deletion and/or non-synonymous mutations in the diterpene synthases (black bars). Further, following separation of *M. marinum* and the MTC, the diterpene synthases were lost from *M. marinum* and several genes inserted, thus disrupting the operon. During later reductive speciation leading to *M. ulcerans*, two of the remaining genes were lost, leaving only the homolog to Rv3383c and flanking segments of the genome. Notably, retention of fully functional diterpene synthase encoding genes (Rv3377c and Rv3378c) is only found in the human-specific pathogen *Mtb*.

The commonly accepted evolutionary scheme for pathogenic mycobacteria indicates that the MTC emerged from a common ancestor, which was most recently shared with *M. canettii*, to create a pool of human and animal pathogens, including *M. bovis* and *M. africanum*, along with *Mtb* (Brosch et al., 2002). Early analysis of the *Mtb* genome isolated a putative five-gene operon involved in isoprenoid biosynthesis at coordinates 3790.85–3799.64 kb and containing loci Rv3377–Rv3383c. This operon was identified as potential horizontal gene transfer products from an exogenous source due to aberrations in GC content within this region (Becq et al., 2007). Rv3377c and Rv3378c are diterpene synthases that produce the immunomodulatory diterpenoid isoTb via cyclization of the diterpenoid precursor GGPP (Nakano et al., 2005a,b; Mann et al., 2009b; Maugel et al., 2010; Prach et al., 2010; Spangler et al., 2010; Hoshino et al., 2011; Mann et al., 2011b; Nakano et al., 2011). In our characterization of the diterpene cyclase encoded by Rv3377c, we noted that this gene was conserved in all four of the *Mtb* strains whose genomes were available at that time, while the *M. bovis* homolog contained an inactivating frameshift mutation that was conserved in the two sequenced strains, along with another that we investigated (Mann et al., 2009a). Based on the wider host range, but reduced infectivity in humans, of *M. bovis*, we then speculated that isoTb might play a role in the highly infectious nature of *Mtb* in humans. Given the current availability of genome sequences from a number of diverse *Mtb* strains (Comas et al., 2010), as well as several members of the MTC and other mycobacteria (Reddy et al., 2009), these were analyzed for the presence of all or part of the isoTb operon. Corresponding sequences could be found not only in the MTC and *M. canettii*, but also the much more distantly related *M. marinum*, although it is not present in the even more distantly related *M. leprae*, suggesting that the isoTb operon was acquired prior to separation of the lineages leading to *M. marinum* and the MTC/*M. canettii*. We further characterized the existing polymorphisms of not only the diterpene synthases [i.e., Rv3377c and Rv3378c, as previously reported (Mann and Peters, 2012)], but also the entire isoTb operon in these mycobacteria. Notably, very few mutations are found within the unique diterpene synthase encoding genes (i.e., Rv3377c and/or Rv3378c) in the sequenced strains of *Mtb* (Comas et al., 2010), with none in the highly infectious Beijing subgroup. By contrast, the other species within the MTC, as well as *M. canettii*, all have accrued frameshift and/or non-synonymous mutations in Rv3377c or Rv3378c. The range of observed mutations in these diterpene synthases is consistent with the current view of mycobacteria evolution, with only a single non-synonymous mutation found in the more recently derived *M. africanum*. By contrast, the more distantly related *M. canettii* contains both non-synonymous and frameshift mutations, wherein the frameshift mutation presumably precludes synthesis of isoTb, much as we previously demonstrated for *M. bovis* (Mann et al., 2009a). In addition, the much earlier diverging *M. marinum* does not contain the diterpene synthase genes at all, although it does have the other genes from this operon. Specifically, *M. marinum* has retained orthologs to the GGPP synthase encoded by Rv3383c, the LytB encoded by Rv3382c, and the pseudo-DXS encoded by Rv3379c, although there also has been an apparent transposon-mediated insertion within the operon. We suggest that this reflects loss of the diterpene synthases, rather than their later addition in the *M. canettii*/MTC lineage, as it is unclear why the other genes, which together produce the diterpene precursor GGPP, would have been assembled together in the absence of the downstream diterpene synthases (and prior to the acquisition of the carotenoid biosynthetic machinery, i.e., as seen in *M. marinum*). In the genome of *M. ulcerans*, which is derived from *M. marinum* (Stinear et al., 2007), the operon has been further reduced, with only the Rv3383c ortholog remaining (**Figure 5**). While this observation is consistent with the previously noted severe genome reduction of *M. ulcerans *(Stinear et al., 2000), *M. marinum* is believed to have undergone parallel evolution from a common ancestor with *Mtb*, but is generally considered to have retained and incorporated more genes, as expected for a more general pathogen, relative to the human host restricted *Mtb* (Stinear et al., 2007). Accordingly, isoTb production seems to be most highly conserved in *Mtb*, despite its comparatively specialized genome, which is consistent with our previous speculation positing a role for isoTb in the highly infectious nature of this human-specific pathogen (Mann and Peters, 2012).

### CONSERVATION OF GGPP SYNTHASES WITHIN THE MYCOBACTERIA

**FIGURE 6:**
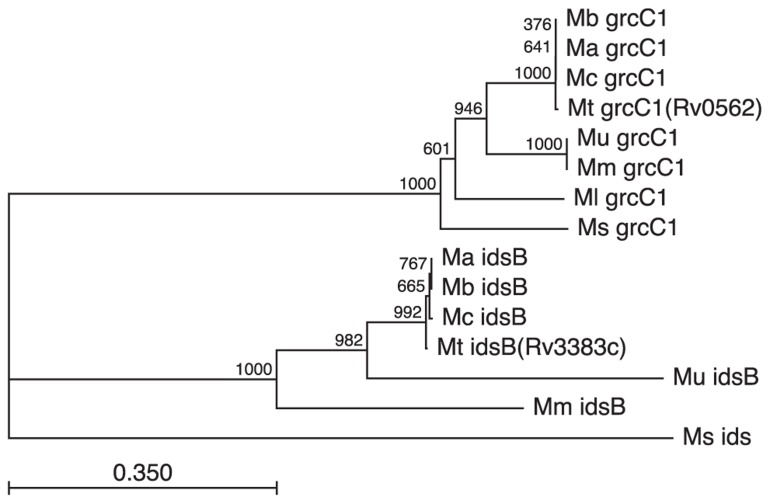
**Conservation of GGPP synthases in the MTC and closely related mycobacteria**. GGPP synthases appear to have assumed additional wider importance in mycobacteria, as evidenced by the retention of Rv3383c/*idsB* despite loss of the remaining isoTb operon. Retention of orthologs for both Rv3383c/*idsB* and Rv0562/*grcC1*, based on both sequence similarity and genome positioning, is found in all sequenced members of the *Mycobacterium* family derived from the last common ancestor of the MTC and *M. marinum*. Species designated by genus and species initials (i.e., Mt, *M. tuberculosis*; Mb, *M. bovis*; Ma, *M. africanum*; Mc, *M. canettii*; Mm, *M. marinum*; Mu, *M. ulcerans*; Ml, *M. leprae*; Ms, *M. smegmatis*) and genes as annotated in the *Mtb* genome (i.e., either *grcC1* or *idsB*), with the next closest *ids* homolog from *M. smegmatis* shown as the outgroup sequence). Not that while many of the listed species do contain other IDS encoding genes, these are relatively distantly related to those shown here.

Although the ability to produce isoTb has not been found and seems unlikely to occur outside of *Mtb*, it is notable that the GGPP producing IDS found in *Mtb* are subject to stronger conservation (**Figure 6**). Strikingly, the GGPP synthase encoding Rv3383c/*idsB* from the isoTb operon was retained even through the reductive evolution process leading to *M. ulcerans* where the remainder of the operon is lost (**Figure 5**). While the presence of a GGPP synthase is likely due to its role in production of carriers involved in cell wall synthesis, the retention of multiple GGPP synthases in these mycobacteria is striking, albeit somewhat puzzling. Rv0562/*grcC1* is likely the ancestral GGPP synthase and is widely found in the family *Mycobacterium*, e.g., an ortholog is present in the fast-growing, non-pathogenic saprophyte *M. smegmatis*. However, those mycobacteria derived from the MTC and *M. marinum* ancestral strain also have retained the additional GGPP synthase encoded by Rv3383c/*idsB*, even upon loss of the other genes in the isoTb operon (e.g., in *M. ulcerans*). In the case of *M. marinum*, this might be due to its production of GGPP-derived carotenoids (Ichiyama et al., 1988), although *M. marinum* also has acquired multiple other IDS homologs. In any case, this rationale does not hold true for other mycobacteria, leaving unclear what selective pressure led to the wider conservation of Rv3383/*idsB* noted here. Interestingly, this specific conservation of dual GGPP synthase homologs across these diverse species of mycobacteria suggests a more significant role for GGPP than previously presumed, consistent with the previous observation of large pools of GGPP-derived geranylgeraniol in *Mtb* cellular extracts (Crick et al., 2000; Mann et al., 2011b). However, it should be noted that the increased sequence divergence of the Rv3383c/*idsB *orthologs in *M. marinum* and *M. ulcerans* also might reflect functional diversification.

## DISCUSSION

*Mycobacterium tuberculosis* is a persistent and deadly human pathogen whose ability to infiltrate and spread within the human immune system has led to intense interest in all aspects of its life cycle (Russell et al., 2010). Isoprenoid metabolites play a variety of roles in *Mtb* and other bacteria, including electron transfer components and cell wall biosynthesis (Daum et al., 2009). At least in the case of *Mtb*, this appears to extend to its pathogenic lifestyle (Dhiman et al., 2004; Kaur et al., 2004; Holsclaw et al., 2008; Mann et al., 2009b). In particular, the bioactive diterpenoid isoTb seems to contribute to early processes in *Mtb* infections, based on both its biological activity (Mann et al., 2009b), and the reduced proliferation of *Mtb *unable to produce isoTb (Pethe et al., 2004). Moreover, specific conservation of the requisite diterpene synthases Rv3377c and Rv3378c in *Mtb* relative to the rest of the species in the MTC (**Figure 5**), has led to the suggestion that isoTb may play a role in the highly infectious nature of *Mtb* in humans (Mann and Peters, 2012), making this a potentially human-specific pathogenic natural product.

The two diterpene synthases required for isoTb biosynthesis were isolated in a screen for genes contributing to the early infection process. However, while the identified Rv3377c and Rv3378c are found in a larger five-gene operon, the remaining three genes were not isolated in this genetic screen, despite its saturating nature (Pethe et al., 2004). Presumably, these three genes originally functioned to increase flux into and through the MEP isoprenoid precursor pathway (i.e., Rv3379c/*dxs2* and Rv3382c/*lytB1*), as well as direct it toward diterpenoid biosynthesis via production of GGPP (i.e., Rv3383c/*idsB*). This would enable production of isoTb by the accompanying diterpene synthases (i.e., Rv3377c and Rv3378c). While the isoTb operon appears to have been acquired via horizontal gene transfer prior to the speciation of *Mtb* and *M. bovis* (Becq et al., 2007), its presence in *M. canettii* demonstrates even earlier acquisition. Moreover, the presence of several of these genes in *M. marinum* suggests that the operon was present in the last common ancestor of the MTC/*M. canettii* and *M. marinum*. In any case, Rv3379–Rv3383c appear to be functionally redundant as none of these genes is essential in *Mtb* (Sassetti et al., 2001, 2003; Sassetti and Rubin, 2003; Joshi et al., 2006), nor do they appear to be absolutely required for isoTb production (Pethe et al., 2004). Consistent with this hypothesis it was previously reported that Rv3379c is an inactive pseudo-gene (Bailey et al., 2002).

It had already been predicted that the *Mtb* genome encodes two functional LytB for the MEP isoprenoid precursor pathway (Camus et al., 2002), and our studies confirm that both Rv1110/*lytB2* and Rv3382c/*lytB1* exhibit such activity (**Figure 2**). Given the non-essential nature of either of these genes indicated by previously reported genetic screens (Sassetti et al., 2001, 2003; Sassetti and Rubin, 2003; Joshi et al., 2006), our results indicate that Rv1110 and Rv3382c are functionally redundant. The essential nature of isoprenoid metabolism, along with the sole use of the distinct MEP pathway for precursor supply in bacteria indicates that the enzymes mediating this process might provide good targets for development of antibacterial agents (Singh et al., 2007; Eoh et al., 2009; Obiol-Pardo et al., 2011). However, it should be noted that, despite their analogous activity, the LytB paralogs characterized here share only 50% AA identity, and such divergence suggests that it may be difficult to simultaneously inhibit both gene products, i.e., to pharmaceutically block this step of the MEP pathway in *Mtb*.

Our results further demonstrate that *Mtb* contains two GGPP synthases, encoded by Rv3383c/*idsB* and Rv0562/*grcC1* (**Table 1** and **Figure 3**). Given the non-essential nature of Rv3383c indicated by previously reported genetic screens (Sassetti et al., 2001, 2003; Sassetti and Rubin, 2003; Joshi et al., 2006), our results suggest that Rv3383c is functionally redundant. By contrast, Rv0562 has been found to be essential in *Mtb* (Sassetti et al., 2003). Its ancestral function presumably was to initiate the production of long chain isoprenylated carriers required for cell wall assembly, as this is derived from GGPP in mycobacteria other than *Mtb* (Besra et al., 1994), and Rv0562 is extremely well conserved in the mycobacterial family (**Figure 6**). However, *Mtb* uses (*Z,E*)-FPP instead of GGPP as the precursor for cell wall isoprenylated carrier biosynthesis (Schulbach et al., 2000). Thus, particularly since Rv0562 is located just downstream of genes encoding other menaquinone biosynthetic machinery in the *Mtb* genome (Camus et al., 2002), we hypothesize that it may instead be required for generation of GGPP for production of this essential electron carrier, presumably as precursor to the known longer chain isoprenyl sidechain (Holsclaw et al., 2008). As an alternative and/or supplemental rationale for the essential nature of Rv0562, our results indicate that this gene encodes a GGPP synthase with more robust and enzymatic activity than that encoded by Rv3383c (i.e., that encoded by Rv0562 can produce GGPP from any length allylic precursor, rather than being limited to FPP as is the IDS encoded by Rv3383c).

In conclusion, our results clarify not only the role of all the genes in the operon associated with isoTb biosynthesis, but also previous metabolic, biochemical, and genetic observations regarding isoprenoid metabolism in *Mtb*, as described above. Nevertheless, there are questions that arise from the results reported here, such as the role of the remaining uncharacterized IDS in the MTC, and the puzzling observation of widespread conservation of Rv3383c/*idsB*, presumably encoding a second GGPP synthase, in mycobacteria, which we hope to address in future studies.

## Conflict of Interest Statement

The authors declare that the research was conducted in the absence of any commercial or financial relationships that could be construed as a potential conflict of interest.
